# Egyptian Society of Cardiology national advisory statement revisiting antiplatelet therapy: insights from E. S. C. 2025 hot-line trials

**DOI:** 10.1186/s43044-026-00769-w

**Published:** 2026-07-21

**Authors:** Sameh Shaheen, Khaled Shokry, Islam Shawky, Hamza Kabil, Gamela Nasr, Mohamad Abd Elghany, Moustafa Mokarrab, Yasser Abd Elhady, Basem Zareef, Haitham Soliman, Mohamad Zahran, Magdy Abd Elhameed

**Affiliations:** 1 Faculty of Medicine, Department of Cardiology, Ain Shams University, Cairo, Egypt; 2 Department of Cardiology, Armed Forces College of Medicine, Cairo, Egypt; 3 Military Medical Academy, Cairo, Egypt; 4 Faculty of Medicine, Department of Cardiology, Al Azhar University, Cairo, Egypt; 5 Faculty of Medicine, Department of Cardiology, Dumyat University, Dumyat, Egypt; 6 Faculty of Medicine, Department of Cardiology, Suez Canal University, Ismailia, Egypt; 7 Faculty of Medicine, Department of Cardiology, Cairo University, Giza, Egypt; 8 Department of Cardiology, Beni-Suef University, Banī Suwayf, Egypt; 9 Department of Cardiology, National Heart Institute, Giza, Egypt; 10 Faculty of Medicine, Department of Cardiology, Fayoum University, Al Fayyum, Egypt

## Abstract

**Background:**

Antiplatelet therapy remains a cornerstone of the management of acute (ACS) and chronic coronary syndromes (CCS). The 2025 European Society of Cardiology (ESC) Congress presented several pivotal Hot-Line trials that challenge established paradigms regarding dual antiplatelet therapy (DAPT) duration, agent selection, and risk-based personalization.

**Purpose:**

This national advisory statement synthesizes the clinical value and implications of 11 major antiplatelet trials presented at ESC 2025 and provides evidence-aligned, context-specific recommendations for cardiovascular practice in Egypt.

**Methods:**

A structured review of ESC 2025 Hot-Line antiplatelet trials was conducted. Evidence was appraised using a predefined grading framework, and national consensus was achieved through a multi-institutional expert panel using modified Delphi methodology. Recommendations were assigned Class of Recommendation and Level of Evidence according to ESC/ACC standards.

**Results:**

The trials addressed antiplatelet therapy in six clinical pathways: ACS, primary PCI in STEMI, cardiogenic shock, post-CABG, complex PCI, and chronic coronary syndrome requiring oral anticoagulation. Key findings include: (1) selective use of shortened DAPT in stabilized ACS; (2) a potential role for P2Y12 monotherapy after 1 month in low-risk, fully revascularized MI; (3) lack of benefit from ultra-early aspirin withdrawal; (4) no superiority of twice-daily aspirin in high-risk ACS; (5) twice-daily clopidogrel for 1 month as a possible alternative to ticagrelor in resource-limited STEMI settings; (6) superior platelet inhibition with intravenous cangrelor in cardiogenic shock without proven clinical superiority; (7) emerging evidence supporting shorter DAPT or aspirin monotherapy after CABG; (8) score-guided DAPT duration in complex PCI; and (9) confirmation that aspirin should not be added to oral anticoagulation in stable coronary disease.

**Conclusion:**

The Egyptian Society of Cardiology supports a more individualized, risk-based approach to antiplatelet therapy, emphasizing ischemic–bleeding balance, stent technology, revascularization completeness, and national resource considerations. Recommendations should be interpreted cautiously given the preliminary nature of some ESC 2025 data.

## Introduction

Antiplatelet therapy has undergone continuous refinement over the past three decades, driven by advances in stent technology, improved understanding of platelet biology, and the development of potent P2Y12 inhibitors. Contemporary management of ACS (acute coronary syndrome) and CCS (chronic coronary syndromes) relies on balancing ischemic protection against bleeding risk, with DAPT (Dual Antiplatelet Therapy) duration and agent selection tailored to patient-specific factors [1–3].

The ESC (European Society of Cardiology) 2025 Congress presented 11 Hot-Line trials that directly challenge or refine current ESC and ACC (American college of Cariology) recommendations. These trials address critical clinical questions: Can DAPT be safely shortened in ACS? Is P2Y12 monotherapy after 1 month appropriate in selected MI (myocardial infarction) patients? Does twice-daily aspirin improve outcomes in high-risk ACS? Can twice-daily clopidogrel rival ticagrelor in STEMI (ST Elevation Myocardial infarction)? Is cangrelor superior to oral agents in cardiogenic shock? Should CABG patients receive prolonged DAPT? Can risk scores guide DAPT duration in complex PCI? Should aspirin be added to oral anticoagulation in CCS?

Given Egypt’s unique healthcare landscape—including variable access to potent P2Y12 inhibitors, cost constraints, heterogeneous stent technology, and high prevalence of diabetes—there is a need for a national advisory that contextualizes these findings. This statement provides a detailed evidence review and practical recommendations for Egyptian cardiologists.

## Methods

*Trial Identification:* All antiplatelet-related Hot-Line trials presented at ESC 2025 were screened. Inclusion criteria were randomized controlled trials (RCTs), adult patients with ACS, STEMI, cardiogenic shock, post-CABG (Coronary Artery Bypass Graft) status, complex PCI (Percutaneous coronary Intervention) or CCS, antiplatelet therapy as a primary intervention, and availability of primary endpoint data. Eleven trials met these criteria.

*Access to trial data:* This was either through direct access to fully published manuscripts in peer reviewed journals or through access to trial data presented during the conference in the hot line sessions, press release or summaries. Peer-reviewed publications were cited when available (Table 1). No unpublished or confidential data were used.

**Table 1:** Eleven trials presented in ESC 2025 and reviewed in this advisory statement

Rial	Publication	Recommendation	Class
DUAL-ACS [6]	ESC Congress 2025	Short-duration DAPT (3 months) may be considered in stabilized ACS patients with low ischemic risk	Class IIa, Level B
TARGET-FIRST [7]	*N Engl J Med*. 2025	Early aspirin discontinuation after 1 month may be reasonable in low-risk MI with complete revascularization using third-generation DES	Class IIa, Level B
NEO-MINDSET [8]	*N Engl J Med*. 2025	Immediate aspirin withdrawal (≤ 4 days) is not recommended	Class III, Level B
ANDAMAN [9]	*Eur Heart J*. 2026	Twice-daily aspirin should not be used routinely	Class III, Level B
TADCLOT [10]	*J Am Coll Cardiol*. 2025	Twice-daily clopidogrel for 1 month may be considered as a cost-effective alternative to ticagrelor in selected STEMI patients	Class IIa, Level B
DAPT-SHOCK-AMI [11]	ESC Congress 2025;	IV cangrelor may be considered when oral absorption is unreliable	Class IIb, Level B
TOP-CABG [12]	*Eur Heart J.* 2025	Three-month DAPT followed by aspirin is reasonable for most ACS patients undergoing isolated CABG	Class IIa, Level B
TACSI [13]	*N Engl J Med*. 2025	Aspirin monotherapy may be preferred in ACS patients at high bleeding risk undergoing isolated CABG	Class IIa, Level B
TAILORED-CHIP [14]	*Eur Heart J.* 2026	Routine escalation–de-escalation strategies in complex PCI are not recommended; standard DAPT should remain the default approach unless individualized risk assessment supports an alternative strategy	Class III, Level B
PARTHENOPE [15]	*J Am Coll Cardiol*. 2025	A score-guided approach to DAPT duration may be considered when institutional systems allow	Class IIa, Level B
AQUATIC [16]	*N Engl J Med*. 2025	Aspirin should not be added to OAC unless there is a compelling indication such as recent stenting	Class III, Level B

*Evidence appraisal:* Each trial was evaluated by a minimum of 2 authors of this manuscript who are 12 academic professors of cardiology from Egyptian universities and major cardiology centers. Each trial was evaluated for: study design (randomization, blinding, control arm); sample size and statistical power; inclusion/exclusion criteria; primary and secondary endpoints; statistical methodology; subgroup analyses; limitations; external validity and applicability to Egypt.

*Consensus development:* To reach a recommendation consensus, the entire author panel arranged for two Delphi rounds with anonymous voting. At least 75% agreement was required for recommendation adoption. All authors had a declaration and management of conflicts of interest.

*Recommendation grading:* Class of Recommendation (I–III) and Level of Evidence (A–C) were assigned according to European society of cardiology standards and guidelines [4, 5].

## Results: evidence review and critical appraisal

Below is a detailed review of all 11 ESC 2025 trials, grouped into 6 clinical pathways.

### Acute coronary syndrome (ACS)

#### DUAL-ACS trial [6]

*Design/population/intervention*: DUAL-ACS was an open-label, randomized, multicenter trial in 5,052 patients with type 1 MI within 12 weeks who were treated with PCI, CABG, or medical therapy and assigned to 3-month versus 12-month DAPT.

*Primary endpoint/results:* All-cause mortality at 15 months was 2.7% with 3-month DAPT versus 3.4% with 12-month DAPT, with numerically lower major bleeding and similar ischemic events.

*Limitations/interpretation:* The trial was underpowered, open-label, and clinically heterogeneous, so the findings support selective abbreviated DAPT in stabilized post-MI patients, particularly those at higher bleeding risk, but not broad routine adoption.

#### TARGET-FIRST trial [7]

*Design/population/intervention*: TARGET-FIRST was a randomized, open-label, multicenter European trial in 1,942 low-risk MI patients who achieved complete revascularization with a Firehawk third-generation DES (Drug Eluting Stents) and were assigned to P2Y12 monotherapy after 1 month versus 12-month DAPT.

*Primary endpoint/results:* P2Y12 monotherapy was non-inferior for net adverse clinical and cerebral events and significantly reduced bleeding.

*Limitations/interpretation:* Because the trial enrolled selected low-risk patients and relied on contemporary stent technology, 1-month DAPT followed by potent P2Y12 monotherapy should be reserved for carefully selected, fully revascularized MI patients with reliable follow-up.

#### NEO-MINDSET trial [8]

*Design/Population/Intervention:* NEO-MINDSET was a randomized, open-label trial in 3,400 ACS patients comparing aspirin withdrawal within 4 days followed by ticagrelor or prasugrel monotherapy versus standard 12-month DAPT.

*Primary endpoint/results:* The composite ischemic endpoint was higher with monotherapy than with DAPT (7.0% vs 5.5%; HR 1.28; 95% CI 0.98–1.68), while major or clinically relevant bleeding was lower (2.0% vs 4.9%; HR 0.40; 95% CI 0.26–0.59).

*Limitations/interpretation:* The early ischemic hazard signal indicate that ultra-early aspirin withdrawal should not be adopted routinely in ACS patients after PCI.

#### ANDAMAN trial [9]

*Design/Population/Intervention*: ANDAMAN was a randomized, double-blind trial in 2,484 high-risk ACS patients, most with diabetes or aspirin-resistance features, comparing aspirin 100 mg once daily versus twice daily.

*Primary endpoint/results:* Twice-daily aspirin did not significantly reduce MACE (Major Adverse Cardiovascular Events) compared with once-daily dosing (7.7% vs 8.8%; HR 0.90; P = 0.42), and major bleeding was similar (1.9% vs 2.1%).

*Limitations/interpretation:* The findings do not support routine twice-daily aspirin dosing and instead reinforce the need for phenotype- or biomarker-guided antiplatelet strategies.

### STEMI primary PCI

#### TADCLOT trial [10]

*Design/Population/Intervention:* TADCLOT was a double-blind randomized trial conducted at National Institute of cardiovascular Disease (NICVD), Pakistan, in STEMI patients undergoing primary PCI, comparing twice-daily clopidogrel with ticagrelor.

*Primary endpoint/results:* Twice-daily clopidogrel was non-inferior to ticagrelor for 30-day MACE, with similar cardiovascular death, stent thrombosis, and major bleeding rates.

*Limitations/interpretation:* The findings are promising for resource-limited settings but require larger external validation before broad substitution for ticagrelor.

### Cardiogenic shock

#### DAPT-SHOCK-AMI trial [11]

*Design/Population/Intervention:* DAPT-SHOCK-AMI was a randomized, multicenter European trial in 605 AMI patients with cardiogenic shock comparing intravenous cangrelor with crushed oral ticagrelor during PCI.

*Primary endpoint/results:* Cangrelor achieved platelet reactivity index < 50% at the end of PCI in 100% of patients versus 22% with ticagrelor, but clinical outcomes at 30 days were not significantly improved and major bleeding was similar.

*Limitations/interpretation:* Cangrelor provides faster and more complete platelet inhibition when oral absorption is unreliable, but absence of proven clinical superiority warrants selective use where available.

### Antiplatelet therapy for ACS patients undergoing CABG

#### TOP-CABG trial [12]

*Design/Population/Intervention:* TOP-CABG randomized ACS patients undergoing isolated CABG to 3-month DAPT followed by aspirin versus 12-month DAPT with ticagrelor plus aspirin.

*Primary endpoint/results:* Three-month DAPT was non-inferior for graft occlusion (10.78% vs 11.19%) and reduced clinically relevant bleeding (8.26% vs 13.19%).

*Limitations/interpretation:* The trial supports CABG-specific antiplatelet protocols and suggests that abbreviated DAPT followed by aspirin monotherapy is reasonable for many ACS patients after isolated CABG.

#### TACSI trial [13]

*Design/Population/Intervention:* TACSI was a large randomized trial in 2,201 ACS patients after CABG across 22 Nordic cardiothoracic centers, comparing aspirin monotherapy with ticagrelor plus aspirin.

*Primary endpoint/results:* MACE at 12 months was similar with DAPT and aspirin alone (4.8% vs 4.6%), while net adverse clinical events and major bleeding were higher with DAPT.

*Limitations/interpretation:* These findings support aspirin monotherapy for most ACS patients after CABG, particularly when bleeding risk is clinically important.

### Complex PCI

#### TAILORED-CHIP trial [14]

*Design/Population/Intervention:* TAILORED-CHIP (Complex High risk PCI) was a randomized trial in 2,018 complex high-risk PCI patients comparing a strategy of ticagrelor plus aspirin for 6 months followed by clopidogrel monotherapy with standard 12-month aspirin plus clopidogrel.

*Primary endpoint/results:* The composite endpoint of death, MI, stroke, stent thrombosis, urgent revascularization, or clinically relevant bleeding was not reduced with the tailored strategy (10.5% vs 8.8%; HR 1.19; 95% CI 0.90–1.58), and bleeding was higher.

*Limitations/interpretation:* Early escalation followed by late de-escalation did not provide net clinical benefit, so standard DAPT remains appropriate for most complex PCI patients.

#### PARTHENOPE trial [15]

*Design/Population/Intervention:* PARTHENOPE prospectively tested DAPT score-guided duration after complex PCI, assigning patients to 3, 6, or 24 months of DAPT based on ischemic risk versus standard 12-month DAPT for all patients.

*Primary endpoint/results:* Tailored DAPT reduced net adverse clinical events, including MI and urgent revascularization, without increasing bleeding.

*Limitations/interpretation:* The trial supports score-guided precision antiplatelet therapy when systems can reliably calculate risk and ensure follow-up, but implementation requires institutional structure.

### Chronic coronary syndrome with oral anticoagulation

#### AQUATIC trial [16]

*Design/Population/Intervention:* AQUATIC was a double-blind, placebo-controlled randomized trial across 51 French centers comparing aspirin plus oral anticoagulation with oral anticoagulation alone in chronic coronary syndrome patients more than 6 months after PCI and at high atherothrombotic risk.

*Primary endpoint/results:* Adding aspirin increased cardiovascular events, all-cause mortality, and major bleeding, while oral anticoagulation monotherapy was safer and at least as effective for ischemic prevention.

*Limitations/interpretation:* Event rates were substantially higher than in prior trials, suggesting a selected high-risk population, but the results strongly support avoiding routine aspirin addition in stable CCS patients already receiving long-term oral anticoagulation unless a compelling indication exists.

## Discussion

### Optimal personalization of antiplatelet therapy in contemporary ACS practice

The 2023 ESC ACS Guidelines [4] endorse a risk-adapted approach to DAPT after PCI. For most ACS patients without major bleeding concerns, 12 months of DAPT with ticagrelor or prasugrel remains a Class I, Level A recommendation. In patients fulfilling ARC-HBR criteria, the ESC supports 1–3 months of DAPT followed by P2Y12 inhibitor monotherapy, based on MASTER DAPT [17] and TWILIGHT [18], earning a Class IIa recommendation. For patients who are not formally HBR but in whom bleeding risk still influences decision-making, earlier de-escalation—supported by TWILIGHT [18] and ULTIMATE-DAPT [19]—is considered reasonable (Class IIb).

The 2025 ACC/AHA/ACEP/NAEMSP/SCAI ACS Guideline [5] similarly advances personalization, granting Class IIa endorsement to P2Y12 inhibitor monotherapy after ≥ 1 month of DAPT in appropriately selected patients.

Recent trials reinforce this shift. TWILIGHT [18] showed that switching to ticagrelor monotherapy after three uneventful months of DAPT halved BARC 2–5 bleeding without increasing MI or stroke. DUAL-ACS [6] and TARGET FIRST [7] extended the safety of shorter DAPT to broader ACS and MI populations. NEO-MINDSET [8] and STOPDAPT-3 [20] demonstrated that aspirin withdrawal should not occur during the vulnerable first month post-PCI, as immediate prasugrel monotherapy increased early stent thrombosis. ANDAMAN [9] further confirmed that intensifying aspirin dosing offers little benefit, supporting biomarker-guided rather than dose-based strategies.

Real-world Egyptian data consistently reinforce TADCLOT’s [10] relevance. Multiple national ACS and PCI registries [21, 22] show that clopidogrel remains the most accessible and widely used P2Y12 inhibitor, driven by cost, availability, and insurance limitations. These same registries [23] document frequent nonadherence and early discontinuation of ticagrelor due to economic barriers, with routine switching from ticagrelor to clopidogrel in public and university hospitals. This evidence demonstrates that clopidogrel’s practical advantages and its noninferiority to ticagrelor in TADCLOT [10] align closely with national treatment patterns, making the trial highly applicable to Egyptian clinical practice and guideline adaptation.

In AMI complicated by cardiogenic shock, DAPT-SHOCK AMI [11] demonstrates that intravenous cangrelor achieves rapid, complete, and reliable P2Y₁₂ inhibition, whereas oral ticagrelor frequently fails due to impaired absorption in shock. The trial shows comparable bleeding risk and signals of early ischemic and mortality benefit, underscoring that timing and reliability of platelet inhibition are critical in this population. Earlier studies [24–26] had already established cangrelor immediate and reversible pharmacodynamic potency, outperforming clopidogrel and modestly reducing early periprocedural ischemic events, albeit with slightly higher non-CABG bleeding. Because major antiplatelet trials—PLATO, TRITON-TIMI 38, and ISAR-REACT 5 [27–29]—largely excluded cardiogenic shock, randomized evidence in this subgroup has been limited. DAPT-SHOCK AMI [11] therefore fills an important gap by confirming that pharmacodynamic failure of oral ticagrelor is common in shock, while IV cangrelor consistently provides full platelet inhibition, even though definitive clinical superiority over potent oral agents has not yet been proven.

In CABG patients, contemporary evidence consistently shows that intensifying or prolonging DAPT offers limited ischemic benefit and increases bleeding. TOP-CABG [12] demonstrated that three months of DAPT followed by aspirin monotherapy maintained saphenous vein graft patency while reducing bleeding. TACSI [13] similarly found that adding ticagrelor to aspirin after CABG in ACS patients increased major bleeding without providing clear ischemic advantage, reinforcing aspirin-based strategies as the preferred approach.

Across the broader CABG literature, POPular-CABG [30] and TARGET [31] also reported no improvement in graft patency with intensified P2Y12 inhibition and documented higher bleeding rates. A 2024 meta-analysis [32] confirmed that while DAPT after CABG may modestly reduce mortality, MACCE, and SVG occlusion, these gains come at the cost of significantly increased major and minor bleeding. Collectively, these trials support aspirin monotherapy as the standard post-CABG antiplatelet strategy, with prolonged or intensified therapy offering little net clinical benefit.

For complex PCI, PARTHENOPE’s score-guided approach [15] is consistent with a *Clinical Consensus Statement of the ESC Working Group on Thrombosis* (2026) [33] recommendations advocating structured risk assessment rather than uniform DAPT durations.

Finally, AQUATIC’s demonstration [16] that aspirin worsens outcomes when added to OAC mirrors earlier trials such as WOEST [34] and AUGUSTUS [35], strengthening the recommendation to avoid combination therapy in stable CCS.

Collectively, these ESC 2025 trials refine—not replace—existing evidence, and their integration into Egyptian practice must consider national epidemiology, resource constraints, and adherence challenges.

### Egypt-specific clinical, social, and system-level considerations

Antiplatelet therapy decisions in Egypt are shaped by a combination of clinical, socioeconomic, and system-level constraints. Access to ticagrelor, prasugrel, and cangrelor is limited, while clopidogrel remains widely available and affordable. This occurs in a population with a disproportionately high burden of diabetes, multivessel disease, diffuse atherosclerosis, and younger MI presentation—typically around 55–58 years—alongside high rates of smoking, obesity, and delayed STEMI arrival. These epidemiologic features increase ischemic risk and reduce the direct applicability of ESC trial populations when considering early DAPT de-escalation [21].

Socioeconomic pressures further influence therapy: out-of-pocket costs frequently lead to premature discontinuation of potent P2Y12 inhibitors, making clopidogrel-based strategies—such as those evaluated in TADCLOT—particularly relevant in resource-limited settings. Access to care varies substantially across public, university, private, military, and police hospitals, with inconsistent availability of potent P2Y12 inhibitors, third-generation DES, and intravascular imaging (IVUS/OCT). These gaps limit the routine implementation of trials that assume contemporary stent technology, optimized implantation, and reliable follow-up [22].

Medication adherence and follow-up are additional challenges, influenced by cost, transportation, work constraints, and limited patient understanding of preventive therapy. These factors increase the risk of premature DAPT discontinuation and argue against aggressive early aspirin withdrawal without safeguards. System-level variability across Egypt—including uneven access to primary PCI, ticagrelor, contemporary DES, platelet function testing, and structured follow-up—means that findings from uniform, high-resource ESC trial environments require contextual adaptation [23].

Applying ESC 2025 trial data in Egypt therefore requires careful selection: shortened DAPT should be reserved for centers with reliable follow-up and contemporary DES; ultra-early aspirin discontinuation should not be routine; twice-daily clopidogrel may be valuable in selected STEMI settings; cangrelor use is limited by availability; CABG patients generally favor aspirin-based strategies; score-guided DAPT requires institutional infrastructure; and aspirin should not routinely accompany oral anticoagulation in stable CCS. These considerations highlight the need for future Egyptian research to define national ischemic and bleeding risk categories, treatment pathways, registry elements, access gaps, and multicenter validation of ESC 2025 recommendations [36].

## Recommendations and clinical algorithm for antiplatelet therapy in Egyptian practice

Clinical decisions should begin with structured assessment of presentation, ischemic risk, and bleeding risk. High ischemic risk includes STEMI, multivessel disease, diabetes, prior stent thrombosis, incomplete revascularization, or complex PCI; bleeding risk should be assessed using ARC-HBR criteria, especially age, anemia, kidney disease, prior bleeding, and need for oral anticoagulation.

In ACS patients treated with PCI, 3-month DAPT may be considered in stabilized, low-ischemic-risk patients treated with contemporary DES. High-bleeding-risk patients may receive 1-month DAPT followed by P2Y12 monotherapy if revascularization is complete and follow-up is reliable, while high-ischemic-risk patients should continue standard 12-month DAPT. Ultra-early aspirin withdrawal and routine twice-daily aspirin are not recommended.

In STEMI patients undergoing primary PCI, ticagrelor remains preferred when accessible and appropriate. Where cost or availability limits ticagrelor use, twice-daily clopidogrel for 30 days may be considered in selected patients, particularly those with high ischemic burden and limited access to potent P2Y12 inhibitors.

In cardiogenic shock, oral P2Y12 absorption is unreliable. Intravenous cangrelor may be used during PCI when available, while crushed ticagrelor should be given early where cangrelor is unavailable.

For ACS patients undergoing isolated CABG, 3-month DAPT followed by aspirin monotherapy is reasonable for most patients. Aspirin monotherapy from the outset is appropriate when bleeding risk is high.

In complex PCI, score-guided DAPT duration may be used when systems allow. Prolonged DAPT may suit high-ischemic-risk, low-bleeding-risk patients, while earlier monotherapy is preferred when bleeding risk is high.

In CCS patients requiring long-term oral anticoagulation, aspirin should not be added unless there is a compelling indication, such as recent stenting, because combination therapy increases bleeding without reducing ischemic events.

All decisions should reflect Egyptian practice realities, including drug access, cost, insurance coverage, adherence, and follow-up reliability. Clopidogrel-based strategies may be necessary when potent P2Y12 inhibitors are unaffordable or unavailable, and aggressive de-escalation should be avoided when follow-up is unreliable.

### Limitations

Some of the ESC 2025 data may not yet be peer-reviewed. Some trials were underpowered or open-label. Applicability varies across Egyptian healthcare settings. Recommendations may require revision as full publications become available. Consensus methodology is not equivalent to formal guideline development.

## Conclusion

**Fig. 1:**
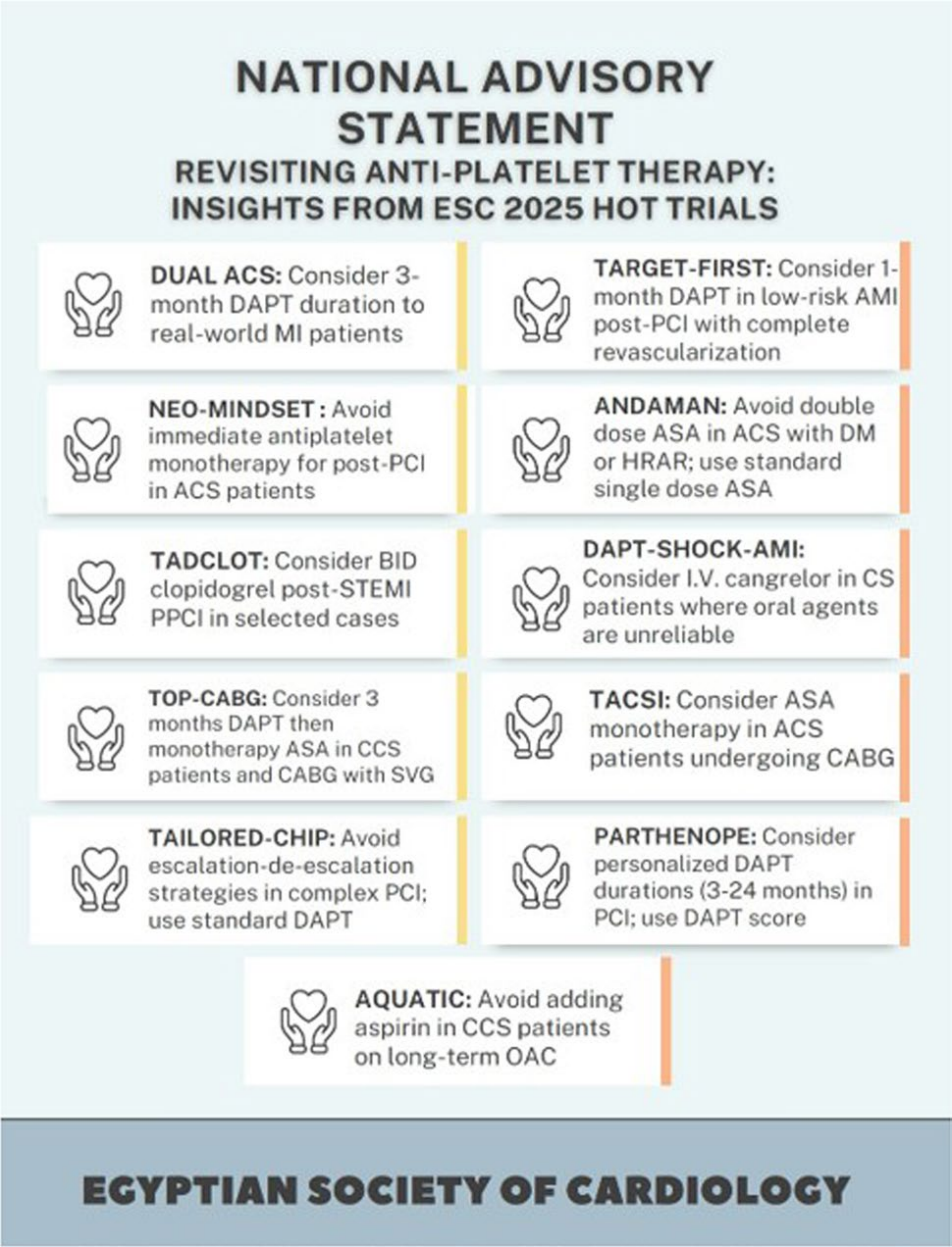
Visual abstract of the Egyptian Society of Cardiology national advisory statement on antiplatelet therapy

ESC 2025 trials support a more individualized, risk-based approach to antiplatelet therapy. This advisory emphasizes cautious interpretation of emerging evidence, prioritization of bleeding–ischemia balance, and adaptation to Egypt’s healthcare realities. Ongoing updates will be required as additional data become available (Fig. 1).

## Data Availability

No datasets were generated or analysed during the current study.

## Abbreviations

ACC: American College of Cariology
ACS: Acute coronary syndrome
CABG: Coronary artery bypass grafting
CCS: Chronic coronary syndrome
CHIP: Complex high-risk PCI
DAPT: Dual antiplatelet therapy
DES: Drug-eluting stents
ESC: European Society of Cardiology
IV: Intravenous
IVUS: Intra-vascular ultra-sound
MACE: Major adverse cardiovascular events
MI: Myocardial infarction
NICVD: National Institute of Cardiovascular Diseases
OAC: Oral anticoagulants
OCT: Optical coherence tomography
PCI: Percutaneous coronary intervention
RCT: Randomized controlled trial
STEMI: ST elevation myocardial infarction
